# Effects of TLR agonists on maturation and function of 3-day dendritic cells from AML patients in complete remission

**DOI:** 10.1186/1479-5876-9-151

**Published:** 2011-09-13

**Authors:** Barbara Beck, Daniela Dörfel, Felix S Lichtenegger, Christiane Geiger, Lysann Lindner, Martina Merk, Dolores J Schendel, Marion Subklewe

**Affiliations:** 1 Department of Internal Medicine III, University of Munich, Campus Großhadern, Munich, Germany; 2 Institute of Molecular Immunology, Helmholtz Zentrum München, German Research Center for Environmental Health, Munich, Germany

## Abstract

**Background:**

Active dendritic cell (DC) immunization protocols are rapidly gaining interest as therapeutic options in patients with acute myeloid leukemia (AML). Here we present for the first time a GMP-compliant 3-day protocol for generation of monocyte-derived DCs using different synthetic Toll-like receptor (TLR) agonists in intensively pretreated patients with AML.

**Methods:**

Four different maturation cocktails were compared for their impact on cell recovery, phenotype, cytokine secretion, migration, and lymphocyte activation in 20 AML patients and 25 healthy controls.

**Results:**

Maturation cocktails containing the TLR7/8 agonists R848 or CL075, with and without the addition of the TLR3 agonist poly(I:C), induced DCs that had a positive costimulatory profile, secreted high levels of IL-12(p70), showed chemotaxis to CCR7 ligands, had the ability to activate NK cells, and efficiently stimulated antigen-specific CD8^+ ^T cells.

**Conclusions:**

Our results demonstrate that this approach translates into biologically improved DCs, not only in healthy controls but also in AML patients. This data supports the clinical application of TLR-matured DCs in patients with AML for activation of innate and adaptive immune responses.

## Background

Acute myeloid leukemia (AML) is the most common acute leukemia in adults, with a poor prognosis and an overall survival (OS) rate of only 23.6% at 5 years (SEER data). Current risk-adapted treatment approaches are determined by several parameters, including cytogenetic characteristics of AML, molecular genetics, age, initial blast count, early blast clearance, and performance status. Although complete remission (CR) rates are high, the majority of patients will suffer from relapse. In the last three decades, various post-remission strategies for elimination of minimal residual disease (MRD) have been developed. The optimal consolidation therapy has not been identified, and patients are offered cytarabine-containing regimens, allogeneic or autologous hematopoietic stem cell transplantation (HSCT), maintenance therapy, and more recently IL-2 in combination with histamine dihydrochloride according to individual risk profile and accompanying morbidity [1,2]. Allogeneic HSCT was shown to provide a potent immunological anti-leukemic effect, with the lowest rate of relapse and a relevant benefit for overall survival in certain age groups [3]. However, this approach is restricted to a subset of patients due to patient-associated morbidity and mortality, donor availability, recipient comorbidities, or age. Clinical vaccination trials with peptides derived from leukemia-associated antigens like proteinase 3 (PR1), Wilm's tumor gene product 1 (WT-1), and the receptor for hyaluronic acid-mediated motility (RHAMM or CD168) have tried to stimulate autologous anti-leukemic T cell responses and have shown promising results regarding immunogenicity and clinical efficacy [4-8].

More recently, an active immunization study with WT-1 RNA-transfected autologous DCs showed immunogenic and anti-leukemic activity while overcoming the HLA-restricted approach of peptide vaccination in AML [9].

DCs are recognized as key regulators of the human immune system, with the ability to induce and maintain primary immune responses as well as tolerance *in vitro *and *in vivo *[10,11]. They have been tested as cellular adjuvants for therapeutic vaccination of solid and hematological malignancies in more than 100 clinical trials since 1996 and proven feasibility and safety. Although immune responses, such as induction of tumor-specific T cells, were observed in many studies, overall clinical response rates remain low. The vast majority of DCs used for clinical trials were derived from autologous peripheral blood monocytes and differentiated with a standard maturation cocktail composed of the cytokines TNF-α, IL-1β, IL-6, and PGE_2_, therefore they lack the capacity to secrete biologically active IL-12(p70) [12]. For optimal T cell activation, it is required that DCs display peptides within MHC molecules as signal 1 and costimulatory molecules as signal 2. In addition, production of IL-12(p70) as signal 3 is desired because of its leading role in promoting T helper 1 (T_H_1) cell polarization and supporting the development of CD8^+ ^cytotoxic T lymphocytes, thereby fostering the appropriate adaptive immune responses needed to combat minimal residual disease and control outgrowth of malignant cells in tumor patients [13,14]. Recently, cocktails containing synthetic TLR agonists emerged as an attractive alternative for the induction of DC maturation [15-18]. TLRs recognize pathogen-derived signals, and stimulation leads to an induction of a T_H_1 immune response via IL-12(p70). Several synthetic TLR agonists could be identified so far. R848 is a low molecular weight synthetic imidazoquinoline compound which activates immune cells via the TLR7/TLR8 MyD88-dependent signaling pathway [17]. Recently, R848 was shown to trigger NF-κB activation in cells expressing murine TLR8 when combined with poly(dT) [19]. CL075 (3M-002) is a thiazoloquinolone derivative that stimulates TLR8 in human peripheral blood mononuclear cells (PBMCs) [20]. It also activates NF-κB and preferentially triggers the production of TNF-α and IL-12(p70). Polyinosine-polycytidylic acid (poly(I:C)) is a synthetic analog of double-stranded RNA (dsRNA), a molecular pattern associated with viral infection. It is composed of a strand of poly(I) annealed to a strand of poly(C) [21]. Poly(I:C) is recognized by TLR3 located mostly in endosomal membranes. It leads to induction of inflammation and long-lasting T cell immunity and matures both mouse and human DCs in a type I IFN dependent fashion [22-24]. The rational for the choice of TLR-agonists was not only based on *in vitro *evidence of effective DC activation but also upon consideration of GMP availability for usage in a clinical trial.

Our present investigation was designed to study the feasibility of a clinical grade 3-day DC generation protocol from non-leukemic monocytes of intensively pretreated AML patients. Novel cocktails containing different synthetic TLR-agonists were used for maturation and were analyzed for their phenotype, costimulatory profile, cytokine secretion pattern, migratory capacity and functional capacity to stimulate NK and T cells.

We were able to show that phenotypically and functionally mature DCs can be generated from heavily pretreated patients with AML as compared to healthy controls. The four different cocktails did not translate into significant differences in the patient and donor cohort. We conclude that this approach is feasible and supports the clinical application of autologous DCs after TLR-maturation in patients with AML for the induction of potent innate and adaptive immune responses.

## Methods

### Source of primary cells

After written informed consent in accordance with the Declaration of Helsinki and approval by the Institutional Review Board of the Ludwig-Maximilians-University (Munich, Germany), peripheral blood samples were obtained from 20 patients with AML in complete remission before initiation of consolidation or maintenance therapy or continuation of the latter. Several patients donated peripheral blood repeatedly. As control group, 25 healthy donors were recruited. The clinical characteristics of the patients are summarized in Table 1.

**Table 1:** Patient characteristics

Patient	Gender	Age (years)	FAB	Karyotype	Molecular mutations		
					
					FLT3-ITD	MLL-PTD	NPM1
# 1	M	64	M2	46, XY	-	-	+
# 2	M	71	M4	46, XY	+	-	+
# 3	M	45	M3v	46, XY, t(15;17)	-	-	-
# 4	F	70	M1	46, XX	-	-	-
# 5	M	69	M4	46, XY	-	-	+
# 6	F	23	M2	46, XX	+	-	+
# 7	M	36	M3	46, XY, t(15;17)	-	-	-
# 8	F	34	M3	46, XX, t(15;17)	-	-	-
# 9	M	51	M3	46, XY, t(15;17)	-	-	-
# 10	F	39	M2	46, XX	-	-	-
# 11	M	48	M2	46, XY	-	-	-
# 12	M	62	M4	46, XY	+^(1)^	-	+
# 13	M	37	M3v	46, XY, t(15;17)	+	-	-
# 14	M	63	M4	46, XY	-	-	-
# 15	M	72	M2	45, X, (-Y)	+	-	+
# 16	F	70	M2	46, XX, t(8;21)	-	-	-
# 17	F	32	M5a	46, XX	-	-	-
# 18	M	55	M4	46, XY	+	-	+
# 19	M	67	M0	51, XY, +9, +10, +11, +13 × 2, +3,	-	-	-
# 20	F	28	sAML	46, XY	-	-	+

Abbreviations: M, male; F, female; FAB, French-American-British classification system

(1) AML #12 = FLT3-TKD mutation

### Cell isolation and generation of mature DCs from PBMCs

In brief, PBMCs were isolated by Ficoll/Hypaque (Biochrom, Berlin, Germany) density gradient centrifugation. Cells were resuspended in RPMI 1640 medium with very low endotoxin (Biochrom, Berlin, Germany), supplemented with 1.5% human serum (pool of AB-positive adult males) (Institute of Transfusion Medicine, Suhl, Germany) - hereafter named DC medium - and allowed to adhere in 6-well plates (Becton Dickinson, Franklin Lakes, NJ, USA) at a concentration of 5 × 10^6 ^cells/mL and a final volume of 2 mL. Non-adherent cells were removed after 90 min of incubation at 37°C and 5% CO_2_.

Immature DCs were generated by culturing the adherent mononuclear cells in medium supplemented with human recombinant GM-CSF (800 IU/mL, Leukine^®^, Bayer, Leverkusen, Germany) and human recombinant IL-4 (580 IU/mL, R&D Systems, Wiesbaden, Germany). After 48 h DCs were additionally cultured with TNF-α at 1100 IU/mL, IL1-β at 2000 IU/mL (R&D Systems), IFN-γ at 5000 IU/mL (Imukin^®^, Boehringer Ingelheim, Ingelheim, Germany), PGE_2 _at 250 ng/mL (Prostin^® ^E2, Pfizer, Borgo San Michele, Italy), and, according to the defined cocktail, R848 at 1 μg/mL or CL075 at 1 μg/mL with or without poly(I:C) at 20 ng/mL (all InvivoGen, San Diego, CA, USA) for 24 hours. The different 3-day DC generation cocktails are summarized in Table 2.

**Table 2:** Cocktails for DC maturation

Cocktail	Inflammatory Cytokines/IFN	Other Additives	TLR Ligands
**C**	TNF-α, IL-1β, IFN-γ	PGE_2_	CL075
**CP**	TNF-α, IL-1β, IFN-γ	PGE_2_	CL075, poly(I:C)
**R**	TNF-α, IL-1β, IFN-γ	PGE_2_	R848
**RP**	TNF-α, IL-1β, IFN-γ	PGE_2_	R848, poly(I:C)

Following concentrations were used in the individual maturation cocktails:

All cocktails contained 1100 IU/mL TNF-α, 2000 IU/mL IL-1β, 5000 U/ml IFN-γ and 250 ng/ml PGE_2_.

Additional additives as follows:

Cocktail **C**: 1 μg/ml CL075

Cocktail **CP**: 1 μg/ml CL075 and 20 ng/ml poly (I:C)

Cocktail **R**: 1 μg/ml R848

Cocktail **RP**: 1 μg/ml R848 and 20 ng/ml poly (I:C)

### Antibodies and multimers

For surface phenotyping, cells were labeled with the following fluorescence-conjugated monoclonal antibodies: CD1a (FITC, clone F7141, Dako Cytomation, Glostrup, Denmark), CD3 (FITC, clone UCHT1, eBiosciences, San Diego, CA, USA), CD4 (APC, clone RPA-T4, BD Biosciences (BD), Heidelberg, Germany), CD8 (FITC, clone HIT8a, BD), CD14 (FITC, clone 61D3, eBiosciences), CD19 (PE, clone HIB19 eBiosciences), CD40 (PE, clone 5C3, eBiosciences), CD56 (PE, clone B159, BD), CD69 (APC, clone FN50, Miltenyi Biotec, Bergisch Gladbach, Germany), CD80 (PE, clone L307.4, BD), CD83 (PE, clone HB15e, eBiosciences), CD86 (FITC, clone 2331 (FUN-1), BD), CD209 (FITC, clone DCN46, BD), CD273 (APC, clone MIH18, BD), CD274 (FITC, clone MIH1, BD), CCR7 (APC, clone FR11-11E8, Miltenyi Biotec), HLA-DR (PE, clone L203, R&D Systems), and corresponding mouse IgG isotype controls were used (FITC, PE and APC, eBiosciences). The percentage of positive cells was determined by subtracting the percentage of cells stained positive with the isotype-matched antibody (set at or below 1%) from the percentage of cells positively stained with the specific antibody. The relative mean fluorescence intensity (MFI) was calculated by dividing the MFI of the measured population by the MFI of cells stained with the isotype-matched antibody. The values are shown as mean ± standard deviation. Multimers were synthesized as previously reported [25]. Specific multimers were used for the peptides A2-CMVpp65 and A2-WT-1 as well as control multimer detecting A2-HIV-gag (kindly provided by D. Busch, Technical University Munich, Munich, Germany). After washing and fixation in FACS buffer containing 2% formaldehyde, the cells were analyzed by flow cytometry using a FACS Calibur instrument (BD Biosciences). Data was analyzed using FlowJo 8 software (Tree Star, Ashland, OR, USA).

### Stability of DC phenotype (wash-out test)

Mature 3-day DCs were harvested and washed 2× with DC medium to completely remove the maturation cocktail. Afterwards, DCs were re-plated in 96 well round bottom wells in DC medium for 24 h. The cells were harvested and stained with the antibodies for various DC surface markers and analyzed by flow cytometry.

### Signal 3 assay of cytokine secretion

Mature DCs were cocultured with CD40L-expressing L929 cells (CD40L transfected mouse fibroblasts, kindly provided by A. Moosmann, Helmholtz Zentrum München, Munich, Germany) as a model system for interaction with activated T cells. Briefly, 5 × 10^4 ^cells/well CD40L-expressing L929 cells were irradiated (180 Gy, cesium source), seeded in a 96-well plate, and allowed to adhere for 24 h at 37°C and 5% CO_2 _in a humified atmosphere. After coculture with 2 × 10^4 ^DCs for 24 h, supernatant was harvested and analyzed by ELISA for the concentration of IL-12(p70) and IL-10 according to the manufacturer's instructions (R&D Systems). Correspondingly, DCs were also cocultured for 24h with allogeneic and autologous non-adherent PBMCs and the supernatant analyzed for IL-12(p70) and IL-10. Non-adherent PBMCs and DCs alone served as a negative control. As applicable, IL-2 (500 IU/mL, Proleukin^®^, Novartis Pharma, Emeryville, CA) or phorbol 12-myristate 13-acetate (PMA, 20 ng/mL, Sigma-Aldrich, Deisenhofen, Germany) or ionomycin (75 ng/mL, Sigma) was used as a positive control.

### Migration assay

After harvesting and washing, DCs were analyzed in a transwell-migration assay. In brief, the lower culture chamber of a 96-trans-well plate (Costar, Corning, USA) was filled with 150 μL migration medium, consisting of VLE-RPMI, 500 IU/mL GM-CSF, 250 IU/mL IL-4 and 1% human serum, with or without the chemokine CCL19 (100 ng/mL, R&D Systems). DCs were seeded in the upper chamber (5 × 10^4 ^cells/well) and incubated for 2 h at 37°C and 5% CO_2 _in a humified atmosphere. DCs from the upper and the lower chamber were collected separately and counted using a Neubauer hemocytometer. Alternatively, the cell number was determined by incubation with CellTiter-Glo reagent according to the manufactor's instruction (Promega) and measured with the luminometer (Victor 3, Perkin Elmer).

### NK cell activation

NK cells were isolated from fresh PBMCs of healthy controls (HCs) by negative selection, according to the manufacturer's instructions (NK cell isolation kit, Miltenyi Biotech). Allogeneic NK cells were stimulated in a 10:1 ratio with DCs by coculture for 24 h in VLE-RPMI supplemented with 10% human serum. As positive control, NK cells were stimulated with IL-2 (500 IU/mL). After 24 h, the supernatant was collected and analyzed for IFN-γ concentration by ELISA (BD Biosciences). For detection of activated NK cell populations, the cocultured cells were stained with CD3, CD56, and CD69 and analyzed by flow cytometry.

### NK cell activation in autologous and allogeneic setting

Non-adherent PBMCs from the same (autologous setup) and an allogeneic donor were cocultured with mature DCs at a ratio of 10:1 with and without the addition of IL-2 (500 IU/mL). As controls, PBMCs alone, PBMCs with IL-2 (500 IU/mL) and PBMCs with PMA (20 ng/mL) and ionomycin (75 ng/mL) were used. After incubation for 19 h, Golgi stop solution consisting of brefeldin A (10 μg/mL, Sigma) and monensin (25 μM, Sigma) was added and allowed to incubate for additional 5 h. The cells were harvested after a total incubation time of 24 h and stained with the surface markers CD3, CD56 and CD69 as well as intracellular cytokine staining for IFN-γ according to the manufactures instructions (BD Biosciences). Cells were analyzed by flow cytometry.

### Proliferation of allogeneic T lymphocytes

CFSE-labeled PBMCs from HCs were cultured in DC medium with allogeneic DCs using PBMCs and DCs at a 10:1 ratio. Following six days of coculture, recovered lymphocytes were analyzed by flow cytometry. The proliferation index was determined by assessment of CD4 positive and negative proliferating cells. PHA (Sigma-Aldrich, Deisenhofen, Germany) and CMV peptide pool 65 (Peptivator, Miltenyi Biotech) served as positive controls, CFSE-labeled PBMCs without DC stimulation as a negative control. Stimulation index (SI) was determined assessing the response to DC stimulation divided by PHA-response (3 μg/mL).

### Induction of antigen-specific T cell responses using HLA-A*02-restricted synthetic peptides

Cytotoxic T lymphocytes (CTLs) were induced using HLA-A*02^+ ^autologous peptide-pulsed 3-day DCs for specific stimulation. In brief, after generation with cocktail RP, DCs were irradiated (35 Gy) and pulsed with 30 μM of the synthetic peptide CMVpp65_495-503 _(NLVPMVATV) or WT-1_126-134 _(RMFPNAPYL) for 2 h at 37°C and 5% CO_2 _in a humified atmosphere. CD8^+ ^T cells were positively enriched from PBMCs using CD8 microbeads and LS columns, as described by the manufacturer (Miltenyi Biotec), and primed with 1 × 10^6 ^DCs in RPMI 1640 containing 10% human serum, 4 mM L-glutamine, 12.5 mM HEPES, 100 IU/mL penicillin and 100 μg/mL streptomycin (Invitrogen, Carlbad, CA) - further named CTL medium. Cytokines were added as follows: IL-2 (50 IU/mL), IL-7 (5 ng/mL, R&D Systems) and IL-15 (10 ng/mL, ImmunoTools, Friesoythe, Germany). After 10 days of culture, CTLs were restimulated with 1 × 10^7 ^irradiated peptide-pulsed autologous PBMCs. Cells were harvested on day 5 after the 2^nd ^restimulation and stained with multimer. In brief, cells were washed in PBS and stained with multimer for 30 min at 37°C and 5% CO_2 _in a humified atmosphere. After washing CD3-APC and CD8-FITC mAb staining was performed for 10 min at 4°C. Cells were fixed with 1% paraformaldehyde and analyzed on a FACS Calibur within 48 h. Unspecific staining with HIV-specific multimer was included.

### ELISPOT assays

ELISPOT plates were coated overnight with unlabelled IFN-γ capture antibody in sterile PBS according to the manufacturer's instruction (BD ELISPOT Set). The plates were blocked for 1 h with sterile PBS containing 1% BSA (Miltenyi) and washed three times with sterile PBS. Single-cell suspensions were plated in RPMI1640 (PAN) with 10% HS and plates were cultured at 37°C with 5% CO_2 _for 24 h, with or without peptide (30 μM). After thorough washing, biotinylated detection antibody was added for overnight incubation. The plates were then washed four times in PBS/BSA containing 0.025% Tween 20. As a tertiary reagent, streptavidin-HRP was added at a 1/1000 dilution in PBS/BSA/Tween 20 and incubated for 2 h, followed by three washes with PBS containing 0.025% Tween 20 and three washes with PBS. The plates were then developed for 3-15 min using AEC substrate. The resulting spots were counted on an AID ELISPOT Reader System specifically designed for morphometric ELISPOT analysis. All results were normalized to 10^6 ^cells and spot number of nonstimulated cells substracted from spot number of stimulated and nonstimulated cells was assessed.

### Statistical analysis

Differences between groups were assessed using a two-sided paired Student's *t *test. A p value < 0.05 was considered statistically significant.

## Results

### DCs can be efficiently generated from AML patients in CR within 3 days using a TLR-containing maturation protocol

Monocytes were obtained by plastic adherence of PBMCs from AML patients in CR and HCs and differentiated into immature DCs using GM-CSF and IL-4 in a fast protocol lasting 48 h [26]. DC maturation was induced after 48 h using four different cocktails summarized in Table 2. Cocktail RP had been used previously to prepare 7 d DCs [27]. After a total culture period of 72 h, cell yields of DCs based on total numbers of seeded cells were analyzed. Recoveries ranged from 8.0 to 10.3%. No significant differences in recovery rates were observed between patients and healthy controls, irrespective of the maturation cocktail used (Table 3).

**Table 3:** Comparison of recovery rates of primary DCs following maturation with different cocktails

	Cocktail	C	CP	R	RP
**Recovery** **(% PBMC)**	**HC-DC** mean ± SD n =	10.3 ± 6.0 8	8.0 ± 5.6 13	8.1 ± 2.9 7	8.4 ± 6.5 12
	**AML-DC** mean ± SD n =	9.9 ± 5.4 14	8.5 ± 6.8 21	9.9 ± 6.3 13	9.1 ± 5.9 16

HC-DC: number of independently generated DC from healthy donors as controls

AML-DC: number of independently generated DC from AML patients

mean: mean, given as percentage of cells determined as quotient of recovered to seeded cells

SD: standard deviation

### TLR-containing cocktails induced a mature and stable surface marker profile on DCs

**Figure 1:**
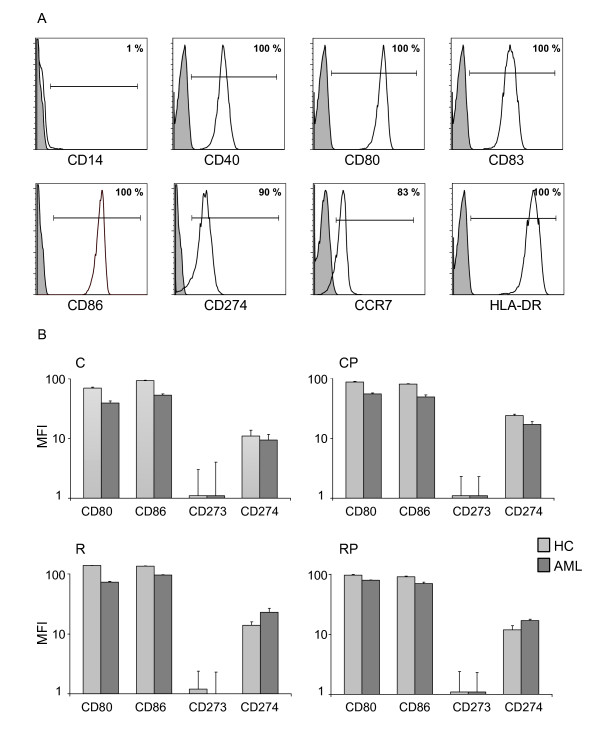
**Costimulatory profile of mature DCs generated with a 3-day protocol**. (A) Shown is the expression of specific surface molecules detected on DCs by flow cytometry in a representative patient sample generated with cocktail RP. (B) Comparison of the expression intensity of the costimulatory molecules CD80 and CD86 and the coinhibitory molecules CD273 and CD274 between HCs and AML patients. The mean fluorescence (MFI) of at least three individual experiments of HC and AML patients were compared using the different maturation cocktails C, CP, R, and RP.

DC populations were analyzed by flow cytometry for expression of characteristic surface markers. In addition to determining the percentages of positive cells compared to isotype control, the mean fluorescence intensity (MFI) was analyzed. A representative patient sample generated with cocktail RP is shown in Figure 1A. Overall, DCs expressed a mature phenotype characterized by high expression of CD83, HLA-DR and low expression of CD14. DCs of patients and HC expressed high levels of CD80, CD86, CD40 and to a lesser degree the inhibitory molecule CD274 and CD273. Statistical analysis did not reveal any significant differences in surface molecule expression of DCs generated with the four different cocktails. Importantly, no substantial differences with respect to surface expression on DCs could be observed between HCs and AML patients except for a slight trend to decreased MFIs of the costimulatory molecules CD80 and CD86 in AML patients. Table 4 summarizes the phenotypic analyses of primary DCs prepared in n ≥ 6 independent experiments from different AML patients in CR in comparison to HCs. The stability of DC phenotype was underlined by re-plating the DCs without any cytokine support for further 24 h. Expression data for the four costimulatory markers directly after maturation and after the 24 hours wash out test are presented in Table 5. No significant changes in DC phenotype for these or any of the other measured antigens (CD14, CD40, CD83, CCR7, HLA-DR) were observed.

**Table 4:** Expression of typical DC surface markers

	CD14						CD40						CD80						CD83					
**% positive cells**	**HD**			**AML**			**HD**			**AML**			**HD**			**AML**			**HD**			**AML**		
C	1	±	1	0	±	0	100	±	0	99	±	1	100	±	0	98	±	3	100	±	0	100	±	0
CP	3	±	4	2	±	4	99	±	1	97	±	9	99	±	1	98	±	4	97	±	9	95	±	10
R	0	±	0	1	±	1	100	±	0	90	±	15	100	±	0	100	±	0	100	±	0	100	±	0
RP	8	±	9	1	±	0	99	±	2	90	±	10	99	±	2	100	±	0	100	±	1	100	±	1
**MFIs**																								
C	2	±	1	1	±	1	22	±	2	27	±	1	108	±	2	61	±	4	41	±	2	35	±	2
CP	2	±	1	2	±	1	19	±	1	15	±	2	87	±	2	55	±	2	36	±	2	27	±	2
R	2	±	1	2	±	1	22	±	2	12	±	2	129	±	1	73	±	2	60	±	1	39	±	1
RP	3	±	2	2	±	1	33	±	2	13	±	2	98	±	2	80	±	2	43	±	2	52	±	1

	**CD86**						**CD274**						**CCR7**						**HLA-DR**					

**% positive cells**	**HD**			**AML**			**HD**			**AML**			**HD**			**AML**			**HD**			**AML**		
C	100	±	0	94	±	14	79	±	44	78	±	40	66	±	37	71	±	22	100	±	0	100	±	0
CP	100	±	1	90	±	25	99	±	2	97	±	5	76	±	25	81	±	14	100	±	0	99	±	2
R	100	±	0	100	±	0	84	±	16	98	±	3	66	±	35	80	±	13	89	±	16	100	±	0
RP	99	±	1	100	±	0	89	±	11	99	±	2	65	±	33	73	±	20	99	±	1	100	±	0
**MFIs**																								
C	87	±	2	46	±	5	10	±	4	8	±	3	5	±	3	6	±	2	278	±	1	327	±	1
CP	80	±	2	49	±	4	24	±	1	17	±	2	8	±	3	18	±	4	273	±	1	171	±	2
R	136	±	1	96	±	2	14	±	2	23	±	4	6	±	2	7	±	2	209	±	2	223	±	2
RP	92	±	2	71	±	3	12	±	2	17	±	1	6	±	3	9	±	2	540	±	1	215	±	2

**Table 5:** Expression of DC surface markers after 24 h without any cytokines (wash-out test)

	CD80						CD86					
**% positive cells**	**0 h**			**24 h**			**0 h**			**24 h**		
C	100	±	1	99	±	1	100	±	0	100	±	0
CP	99	±	1	99	±	1	100	±	1	100	±	0
R	100	±	1	99	±	1	100	±	1	100	±	0
RP	99	±	2	96	±	4	99	±	1	99	±	0
												
**MFIs**												
C	70	±	2	69	±	2	93	±	2	121	±	1
CP	76	±	2	67	±	2	84	±	2	119	±	1
R	70	±	2	62	±	1	112	±	2	91	±	2
RP	72	±	2	46	±	2	108	±	2	108	±	1

	**CD273**						**CD274**					

**% positive cells**	**0 h**			**24 h**			**0 h**			**24 h**		
C	3	±	3	11	±	7	98	±	2	92	±	6
CP	4	±	3	10	±	8	98	±	1	93	±	5
R	5	±	4	7	±	9	93	±	10	86	±	9
RP	5	±	5	15	±	14	91	±	10	89	±	8
**MFIs**												
C	1	±	2	1	±	1	11	±	3	10	±	1
CP	1	±	1	1	±	1	18	±	2	10	±	1
R	1	±	1	1	±	2	13	±	2	8	±	2
RP	1	±	1	1	±	2	13	±	2	10	±	1

### DCs from AML patients and HCs displayed a prominent costimulatory profile

TLR signaling increased the functional potential of DCs for priming T cells, but coinduction of potentially negative immunoregulatory capacities may impair effector T cell generation. Therefore, we examined the expression of the following members of the B7 family: CD80 (B7.1), CD86 (B7-2), CD273 (B7-DC/PD-L2) and CD274 (B7-H1/PD-L1). A dominance of CD80 and CD86 compared to CD273 and CD274 was observed. Figure 1B shows MFI values for DCs matured with cocktails R, RP, C, and CP. All generated DCs express a positive ratio of the positive costimulatory molecules CD80 and CD86 to the negative costimulatory molecules CD273 and CD274. No significant differences in MFI of these costimulatory molecules were observed using the four different maturation cocktails (sample size: CD80/CD86/CD273/CD274 in HC: C: n = 10/10/4/8, CP: n = 16/16/3/7, R: n = 13/11/4/13, RP: n = 11/9/3/10; in AML patients: C: n = 10/10/4/9, CP: n = 14/14/3/10, R: n = 10/7/4/10, RP: n = 8/6/3/6). More importantly, we did not observe a significant difference in costimulatory expression pattern between DCs generated from HC and AML patients.

### IL-12(p70) and IL-10 secretion by DCs

**Figure 2:**
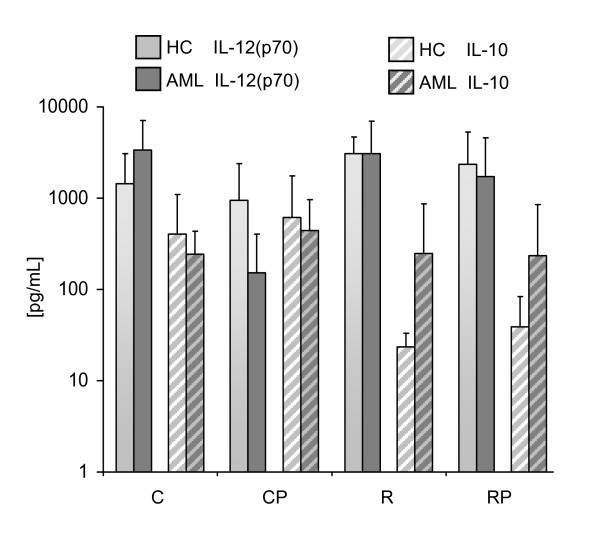
**Cytokine secretion assessed by signal-3 assay**. CD40L-transfected mouse fibroblasts were used as stimulators for DCs mimicking T cell contact. Supernatants were collected after 24 h of coculture. Cytokines were measured by standard ELISA. Independent experiments were performed with a number of n = 4-12 donors for C, CP, R, and RP in HCs and AML patients.

In contrast to the maturation cocktails used for generation of DCs in clinical trials so far, our primary goal was the identification of a TLR-containing maturation cocktail that generates DCs in AML patients that secrete high amounts of bioactive IL-12(p70) while producing no or only low levels of IL-10. To investigate whether DCs retain this capacity upon encounter with T cells in lymph nodes, a signal 3 assay was performed. DCs were cocultured with a murine fibroblast cell line that expresses human CD40L and thereby mimics the encounter of DCs with CD40L^+ ^expressing T cells. IL-12(p70) and IL-10 release was determined after coculture for 24 h by standard ELISA for a varying number of patients and HC due to different amounts of available blood and PBMC recovery (HCs: C: n = 7, CP: n = 17, R: n = 4, RP: n = 12; AML: C: n = 5, CP: n = 11, R: n = 9, RP: n = 12). The results are shown in Figure 2 as mean values of IL-12(p70) and IL-10 in picograms per milliliter. Unstimulated DCs from HC as well as AML patients did not secrete any relevant amounts of IL-12(p70) or IL-10 (< 15 pg/mL). Because IL-10 counteracts IL-12(p70), we calculated the ratio of IL-12(p70)/IL-10 for all DC populations: HC: C: 6.1 ± 33.5 CP: 2.3 ± 16.4; R: 126.8 ± 2.5 and RP: 28.4 ± 6.7; AML patients: C: 16.7 ± 4.6; CP: 0.5 ± 10.3; R: 12.5 ± 25.5 and RP: 7.8 ± 17.6. Inter-individual variations were noted in the capacity of DCs derived from different donors to produce these cytokines, but no significant differences between AML patients and HCs. To validate the significance of the signal 3 assay using human CD40 L transfected fibroblasts, we cocultured mature DCs with autologous and allogeneic T cells for 24 h. For HC and AML patients we observed a consistent secretion of IL-12(p70) and no detectable level of IL-10 using the four different cocktails. As expected, we measured higher amounts of IL-12 in the supernatant media of DC coculture with allogeneic T cells versus autologous T cells (data not shown).

### DCs showed chemotaxis to CCL19 signals

**Figure 3:**
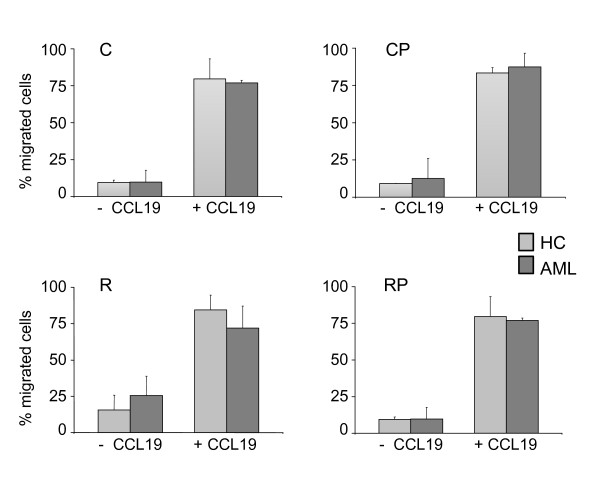
**Migratory capacity of DCs to CCR7 ligand**. Depicted are data of at least two independent trans-well migration assays using CCL19 (100 ng/mL) as chemoattractant for DCs generated with cocktail C, CP, R and RP. Shown are the mean values ± standard deviations.

For the successful induction of immune responses, *in vivo *homing of DCs to secondary lymphoid organs is crucial. CCR7-mediated signals control this migration process and subsequently their positioning within defined functional compartments. To address the migratory capacity of DCs from AML patients, we conducted a standard trans-well migration assay in the presence of the CCR7 ligand C-C-motif chemokine 19 (CCL19). As illustrated in Figure 3, DCs generated with all four cocktails showed very little spontaneous migration in the absence of chemokines. DCs from HCs and AML patients migrated efficiently towards CCL19. The data suggests that DCs from AML patients had comparable migration capacity towards chemokines important for homing to secondary lymphoid organs compared to healthy donors.

### DCs prepared using TLR7/8 agonists strongly activate NK cells

**Figure 4:**
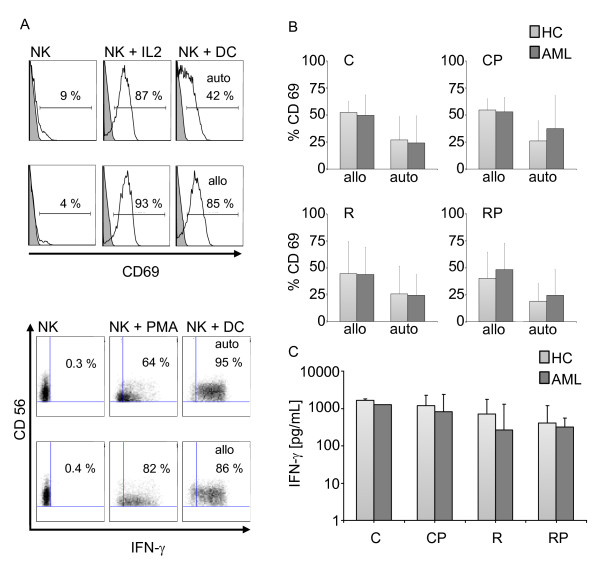
**Activation of NK cells by DCs**. Allogeneic and autologous non-adherent PBMCs were cocultured with DCs at a ratio of 10:1 for 24 h. Cells were stained for CD3, CD56, CD69 and IFN-γ expression and analyzed by flow cytometry. (A) Shown is the percentage of CD69 expression on CD3^-^/CD56^+ ^gated NK cells. Representative histograms of unstimulated NK cells, IL-2 (500 IU/mL) activated NK cells, DCs + autologous NK cells (upper panel) and DCs + allogeneic NK cells (lower panel) of one AML patient are shown (cocktail R was used for DC maturation). The dot blots below show the corresponding intracellular IFN-γ staining, with PMA/ionomycin serving as a positive control. (B) Summary of the percentage of CD69 expression on CD3^-^/CD56^+ ^NK cells after coculture with allogeneic and autologous NK cells from HC and AML patients. (C) Production of IFN-γ after coculture of DCs and isolated NK cells was assessed by ELISA. DCs were generated and cocultured with allogeneic NK cells at a ratio of 1:10 for 24 h. Supernatant was analyzed by standard ELISA for IFN-γ. The comparison of HC and AML patients is shown using the four different maturation cocktails. As a positive control NK cells were stimulated with IL-2.

The functional interaction of DCs and NK cells plays a crucial role in regulating the link between innate and adaptive immune responses. The maturation process of DCs is accompanied by a progressive accumulation of a variety of cytokines, including IL-12(p70), which induces NK cell activation [28]. We addressed the stimulating capacity of DCs matured with different TLR-containing cocktails by examining the upregulation of the activation marker CD69 (Figure 4A/B) and the production of intracellular IFN-γ (Figure 4A/C) upon coculture with autologous and allogeneic NK cells. DCs were able to activate NK cells after 24 h of coculture in an autologous as well as in an allogeneic setting. A representative example is shown in Figure 4A using CD69 upregulation on CD3^-^/CD56^+ ^NK cells as well as production of IFN-γ as a readout. A summary of the results is shown in Figure 4B demonstrating potent activation of autologous and allogeneic NK cells by DCs from HC and AML patients using the four different cocktails (sample size for HC allo: C/CP/R/RP n = 6/8/9/6; HC auto n = 4/4/6/6; AML allo: n = 9/10/13/10; AML auto: n = 4/4/7/6. These results were supported by similar findings after coculture of DCs with isolated NK cells (negatively selected). Again, using a purified NK cell population we were able to detect high amounts of IFN-γ in the supernatant after 24 h coculture. We did not see any significant differences between HC and AML patients using the four different cocktails. As the process of negative selection of NK cells requires higher numbers of leukocytes at the beginning, this experimental setting was only done using healthy donors as the source of NK cells, hence only in an allogeneic setting (Figure 4C).

### DCs prepared using TLR7/8 agonists stimulate allogeneic CD4 T cells

**Figure 5:**
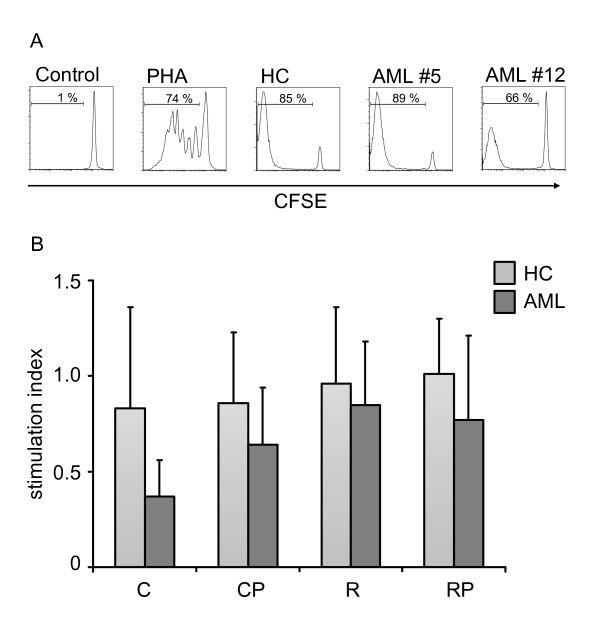
**Activation of allogeneic T cells by DCs**. PBMCs from healthy unrelated donors were stained with CFSE and cocultured with DCs at a ratio of 10:1. After 6 days, proliferation was assessed by flow cytometry. (A) Representative examples of unstimulated cells and proliferating cells are shown in the presence of PHA (3 μg/mL) and after coculture with DCs generated from HCs and two AML patients (AML #5, AML #12). (B) Stimulation index of DCs generated from HCs and AML patients with cocktail C, CP, R, and RP (n = 3-9) are summarized as mean ± SD.

CFSE-labeled PBMCs from HCs were cultured with allogeneic DCs from HC and AML patients at a 10:1 ratio. Following six days of coculture, recovered lymphocytes were analyzed by flow cytometry. PHA served as a positive control, CFSE-labeled PBMCs without DC stimulation as a negative control. Stimulation index (SI) was determined by assessing the response to DC stimulation divided by PHA-response (Figure 5A/B). We could observe excellent stimulatory capacity of allogeneic T cells by DCs generated from AML patients with no significant difference between the four different maturation cocktails. Importantly, no significant difference between AML patients and HCs could be observed (n = HC (C/CP/R/RP): 3/9/5/9; AML (C/CP/R/RP): 3/5/8/8).

### Generation of CMVpp65- and WT-1-specific CTLs using 3-day DCs

**Figure 6:**
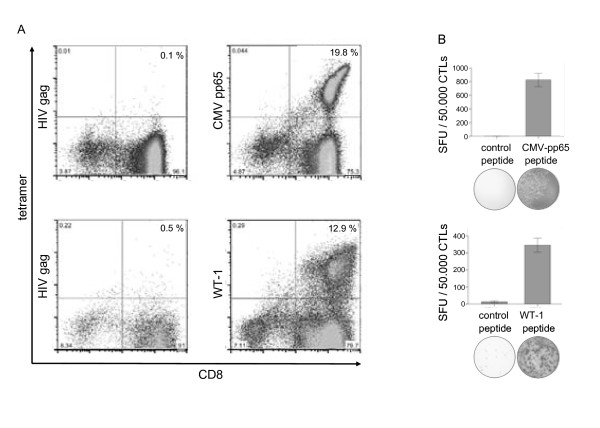
**Induction of CMVpp65 and WT-1 specific T-cell response with peptide loaded 3-day DCs**. (A) Percentage of CMVpp65- and WT1-specific CD8 T cells was determined by staining with anti-CD8 and the appropriate HLA-matching multimeric complex. As a negative control, a tetramer specific for an HIV-specific peptide was included. Gates were set on the lymphocyte population and on CD3 positive cells. Results are given as % CD8/tetramer double-positive cells. DCs used for initial stimulation were generated using the RP cocktail. One representative experiment is shown. (B) The functional activity of the CTL is shown by IFN-γ production response to peptide loaded target cells in the ELISPOT assay.

CMVpp65- and WT-1-specific T cells were generated by stimulation with CMVpp65_495-503 _(NLVPMVATV) and WT-1_126-134 _(RMFPNAPYL) *in vitro *using peptide-pulsed 3-day DCs with cocktail RP. After two restimulations with peptide-pulsed PBMCs, antigen-specific T cells were quantified using multimer technology. A significant expansion of CMVpp65-specific T cells (19.8% of total CD8 T cells) and WT-1-specific T cells (12.9%) could be detected. (Figure 6A). To investigate the functional specificity of T cell lines, ELISPOT for IFN-γ secretion was performed. Peptide-specific IFN-γ secretion corresponding to multimer-positive peptide-specific T cells was observed (Figure 6B).

## Discussion

DC-based vaccination has been intensively investigated since the first trial in patients with B cell lymphoma in 1996 [29]. Although DC-based immunotherapies have induced immunologic responses in the majority of trials, thus far only a limited number of clinical responses have been observed. It remains unclear why some patients respond to DC-based immunotherapy and others do not, but it has been suggested that the current protocols used to generate mature DCs may not result in optimal T_H_1 and NK cell responses. Also, these trials were mainly performed in late-stage immunosuppressed cancer patients as a result of extensive radiation, chemotherapy and/or large tumor burdens [30]. The unique capacity of DCs to activate and expand different arms of cell-mediated resistance, such as NK, NKT, B and T cells, each of which recognizes different alterations in cancer cells, could be ideally exploited using IL-12(p70) secreting DCs with a positive costimulatory profile.

We therefore, evaluated the generation of such DCs from monocytes of AML patients in complete remission following intensive chemotherapy. As a prerequisite, monocyte counts of AML patients were analyzed before consolidation and maintenance therapy. The mean number of leukocytes and monocytes was comparable to HCs, making the generation of mature DCs feasible from these patients.

The discovery that adjuvants can stimulate innate immunity by interacting with specialized pattern recognition receptors (PRRs), including TLRs, opened the door for the inclusion of the respective agonists in DC maturation cocktails [31]. TLRs allow the recognition of a range of pathogens and lead to *de novo *transcription and secretion of cytokines and chemokines, enhanced antigen presentation capacity, and superior migration to lymphoid tissues. In humans, expression of TLR1 to 6 and TLR 8 were described in monocyte-derived DCs [32-35]. The signaling pathways associated with ligation of each of these TLRs are not identical; therefore distinct biologic responses are initiated upon receptor ligation [36]. The availability of different synthetic ligands for TLRs, like imiquimod (R837), resiquimod (R848), S-27609, CL097, CL075 (3M-002), CL087, or loxoribine, has stimulated research to use TLR agonists in maturation cocktails. For example, combinations of the TLR3 ligand polyinosinic-polycytidylic acid (poly(I:C)) and the TLR7/8 ligands R848 or CL075, supplemented with PGE_2_, yielded DCs with both high migratory capacity and high IL-12(p70) production upon T cell encounter [37,38]. TLR-mediated maturation of *ex vivo-*generated human DCs may thus be used to improve immunologic and clinical responses in DC vaccination of cancer patients. Furthermore, it was shown that 3-day DCs were as effective as conventional 7-day DCs in stimulating primary, antigen-specific T_H_1 immune responses. This fast generation not only reduces labor, cost and time required for DC development, but also represents a model more closely resembling DC differentiation from monocytes *in vivo *[26].

We tested two different TLR7/8 agonists, with or without the TLR3 agonist poly(I:C), for their potency to induce mature DCs in AML patients in comparison to HCs. Phenotypically, DCs from AML patients showed high CD83, low CD14 and a slightly reduced expression of the costimulatory molecules CD80 and CD86 in combination with a similar expression intensity of CD274 and CD273. The predominance of positive costimulatory molecules over inhibitory molecules translates into potent T cell stimulation, as demonstrated by Selenko-Gebauer et al. [39]. In accordance, we also observed a potent allostimulatory capacity of TLR-matured DCs from AML patients as well as healthy controls.

Successful anticancer immunotherapy requires strong T_H_1-polarizing immune responses as well as NK cell responses [40-43]. In a recently published clinical phase I trial using RNA-transfected DCs from AML patients, a significant correlation could be seen between clinical responses and the induction of high numbers of activated NK cells [9]. Thus, DCs are required that not only express positive costimulatory markers, but also secrete high amounts of bioactive IL-12(p70). This DC-derived cytokine constitutes signal 3 of T cell activation and determines outcomes of T cell differentiation as well as NK cell activation [40,41,44]. Previous reports on DC generation from monocytes of AML patients in remission have used the classical cytokine cocktail containing TNF-α, IL-1β, IL-6 and PGE_2 _for DC maturation [12,45]. This protocol generates immature DCs over 5-7 days before the maturation cocktail is added for the final 48 hours. As previously described by others, DCs generated by this cocktail do not produce measurable IL-12(p70) [27,37]. In the published literature on DC generation from AML patients there is no data regarding secretion of DC-derived cytokines nor is there information regarding DC activation of NK cells [45,46]. This report is the first to show that a TLR- containing maturation cocktail is able to generate DCs from AML patients in only 3 days with high potential for induction of both T- and NK-cell responses. We demonstrated high secretion of DC derived IL-12(p70) which translated into strong NK cell activation, as shown by CD69 upregulation and IFN-γ secretion by the CD3^-^/CD56^+ ^NK cell subpopulation. The strong effects on DC phenotype and function were primarily induced by TLR 7/8 ligation as the addition of the TLR3 ligand poly(I:C) did not result in any significant changes in the expression pattern of costimulatory molecules or IL-12(p70) secretion. This finding may be explained by the low poly(I:C) concentration used in our cocktails which is up > 100 fold lower compared to other published reports [47,48]. The significant lower concentration of poly(I:C) was chosen due to the observations that poly(I:C) at a high concentration prevents DCs from being able to express protein after loading with exogeneous RNA, while the lower concentration is still sufficient for DC activation [27]. This is of utmost importance for our planned clinical phase I/II trial, as we will use RNA-transfected DCs for active immunotherapy in AML patients.

A promising target antigen for immunotherapeutic approaches in AML is WT-1, which is generally overexpressed in the majority of AML cases [49]. Several clinical trials using active immunotherapeutic strategies have targeted WT-1, so far with promising immunological and clinical responses [8,50]. We tested our DCs for their potential to expand WT-1-specific, CD8^+ ^T cells. Comparative data was obtained against the immunodominant CMVpp65 protein and T cell responses were analyzed by multimer and ELISPOT for IFN-γ. Equally effective expansion of WT-1 - and CMVpp65- specific T cells could be observed. Our data demonstrate that TLR-containing maturation cocktail can generate DCs from AML patients with phenotypic and functional characteristics that are highly desirable for use in a clinical trial setting.

A clinical phase I/II study is currently in preparation using non-leukemic monocytes for generation of autologous DCs in AML patients in complete remission. These DCs will be transfected with RNA encoding leukemia-associated antigens for induction of leukemia-specific immune responses in vaccinated patients. Through their secretion of IL-12(p70), the DCs will favour type 1 polarization of T cells while allowing NK cell activation to be initiated at the same time. We believe that these DCs are highly suitable for application in cancer immunotherapy.

## Conclusions

To our knowledge, this is the first report in which a TLR-containing maturation cocktail has been used for generation of young, 3-day, DCs from AML patients in remission in comparison to HC. We found that mature DCs prepared with the TLR7/8 agonists, R848 or CL075, displayed phenotypes and functions suitable for antitumor vaccine development with no significant differences between AML and HCs and among the different TLR-containing cocktails. As the manufacturing protocol is easily adaptable to GMP requirements, we propose the implementation of our protocol in a clinical vaccination trial in AML patients in complete remission for eradication of minimal residual disease.

## Competing interests

DJS and the Helmholtz Zentrum München has pending patents on the maturation cocktails tested in these studies.

## Authors' contributions

BB and DD contributed equally to this manuscript. BB carried out the studies, performed statistical analyses and helped to draft the manuscript. DD carried out part of the studies and drafted the manuscript. FL, CG, MM and LL carried out part of the studies. DJS participated in the design of the study. MS conceived and designed the study and coordinated and drafted the manuscript. All authors read and approved the final manuscript.
